# Pregnancy-Related Carpal Tunnel Syndrome

**DOI:** 10.7759/cureus.94652

**Published:** 2025-10-15

**Authors:** Filip Georgiew, Jakub Florek, Adam Bębenek, Pawel Florek, Grzegorz Sobanski

**Affiliations:** 1 Faculty of Health Science, University of Applied Science in Tarnów, Tarnów, POL; 2 Department of Orthopaedics and Traumatology, Rydygier Hospital, Brzesko, POL; 3 Department of Neurosurgery, St Lucas Hospital, Tarnów, POL; 4 Department of Physical Medicine and Rehabilitation, Reha Medica Medical and Rehabilitation Center, Tarnów, POL

**Keywords:** carpal tunnel syndrome, conservative treatment, hand, pain, pregnancy

## Abstract

Carpal tunnel syndrome (CTS) is a common musculoskeletal disorder during pregnancy. Symptoms are often mild, and only a minority of affected women seek medical attention. Although symptoms typically resolve after delivery, they may persist through breastfeeding or beyond in some cases. Given this course, conservative management is recommended. This article aims to review pregnancy-related carpal tunnel syndrome (PRCTS), with particular emphasis on indications for available conservative treatment modalities. In this narrative review, a computer-based search was conducted on PubMed, Scopus, The Cochrane Library, and Web of Science databases for the relevant literature. Studies published between 2005 and 2025 were included. The search strategy included a combination of the following search terms: carpal tunnel syndrome, pregnancy, conservative treatment, hand, and pain. No specific limitations were imposed on study selection criteria. In PRCTS, the primary treatment goal is to reduce intracarpal pressure. First-line measures include wrist immobilization, pharmacologic and anti-edema therapies, and techniques to mobilize or stretch the transverse carpal ligament. Initiation of pharmacologic treatment during pregnancy should be carefully weighed because of potential maternal and fetal adverse effects. Commonly used physiotherapy procedures in the treatment of CTS, including high-intensity laser therapy and extracorporeal shock wave therapy, are contraindicated during pregnancy. Therapeutic ultrasound and phonophoresis are generally avoided or used only with caution. Surgical intervention is infrequently required and is reserved for cases with severe symptoms and functional impairment, failure of nonsurgical measures, and electrophysiological evidence of marked nerve compression.

## Introduction and background

Wrist and hand pain is a common symptom reported by pregnant women. Data provided by Kesikburun et al. show that up to 33.2% of women complained of musculoskeletal pain in the hand and wrist in pregnancy [1]. Balik et al. studied the hand and wrist problems in 383 pregnant females. The authors reported that according to the Boston Carpal Tunnel Questionnaire, 258 (67.4%) pregnant women were symptomatic. Nonspecific symptoms were observed in 138 (36%), tendinitis in 80 (20.9%), and carpal tunnel syndrome in 39 (10.2%) [2]. Data cited by Afshar and Tabrizi show that carpal tunnel syndrome (CTS) is a common musculoskeletal condition during pregnancy, second only to lower back pain [3]. CTS results from compression of the median nerve within the carpal tunnel--a narrow passageway in the wrist--leading to pain, numbness, and weakness in the hand. During pregnancy, fluid retention and hormonal changes can exacerbate this compression. A literature review clearly shows that pregnancy emerges as a significant period of risk for the development of CTS, with reported prevalence rates among pregnant women ranging from 0.23% to 70%, depending on the diagnostic method. It is understandable that authors basing the diagnosis on electrophysiological findings obtain lower incidence rates than those based solely on symptoms or clinical findings. Due to the lack of a single standard for the diagnosis of CTS, comparison of epidemiological data is difficult. Nevertheless, the results presented in Table 1 demonstrate that pregnancy-related carpal tunnel syndrome (PRCTS) is a frequently diagnosed pathology in pregnant women.

**Table 1:** Incidence rates of pregnancy-related carpal tunnel syndrome PRCTS: pregnancy-related carpal tunnel syndrome; CTS: carpal tunnel syndrome

Authors	PRCTS Incidence Rates
Balik et al. [2]	383 pregnant females at ≥28 weeks gestation - 39 (10.2%)
Padua et al. [4]	7% - 43% electrophysiological CTS; 31% - 62% of patients were reported to have self-reported CTS
Yazdanpanah [5]	1508 pregnant females; 51 - 64 hands (3.4%) with clinical and electrophysiological CTS
Rozali et al. [6], Sapuan et al. [7]	333 pregnant females in the third trimester – 82 (24,6%)
Mora et al. [8]	368 pregnant females in the third trimester – 102 (27,7%)
Afaq et al. [9]	75 pregnant females – 36 (48%)
Meems et al. [10]	639 pregnant females – 219 (34%)
Oliveira et al. [11]	482 pregnant females – 111 (23.3%)
Khan et al. [12]	200 pregnant females – 60 (30%)
Hamoda et al. [13]	19%
Ferraz et al. [14]	2 - 70%
Yaseen et al. [15]	2% - 5%,
Cîmpeanu et al. [16]	0.23% - 70%

Walshe first reported the association between pregnancy and CTS in 1945 [17]. Jones later described cases treated with diuretics in 1953 [18]. Symptoms most often emerge in the third trimester [3,15,16]; they are usually mild and only a minority of affected women seek medical attention. Although symptoms typically resolve after delivery, they may persist through breastfeeding or longer in some patients: Pauda et al. found that symptoms persisted in over 50% at one year and in ~30% at 3 years postpartum [4]. This article aims to review PRCTS, with particular emphasis on indications for available conservative treatment modalities, which is preferred because symptoms often subside after childbirth.

## Review

Methods

In this narrative review, a computer-based search was conducted on PubMed, Scopus, The Cochrane Library, and Web of Science databases for the relevant literature. Studies published between 2005 and 2025 were included. The search strategy included a combination of the following search terms: carpal tunnel syndrome, pregnancy, conservative treatment, hand, and pain. No specific limitations were imposed on study selection criteria. To ensure comprehensive coverage, relevant journals and conference proceedings were manually searched to supplement the database search. Additionally, the reference lists of retrieved articles were meticulously examined to identify any additional relevant publications. Finally, 36 articles qualified for the review. Additionally, in the part of the article covering conservative treatment methods, we also relied on our practical knowledge and experience regarding the management of patients with PRCTR.

Etiology

Several factors increase the risk of PRCTS in pregnant and postpartum women. First, plasma volume expansion and fluid retention lead to edema and reduced venous return; tissue swelling within the carpal tunnel raises intracarpal pressure and precipitates median nerve compression [3,9,15,19,20]. Meems et al. and Yaseen et al. identify late-pregnancy fluid accumulation as a key exacerbating factor for PRCTS symptoms [10,15]. These changes are mediated by altered hormonal milieu, notably elevated estrogen, progesterone, aldosterone, cortisol, and prolactin concentrations [9,16,20]. Second, increased nerve sensitivity to ordinarily subthreshold stimuli (including pressure) may contribute to an effect analogous to that seen in diabetic neuropathy [19]; gestational diabetes may therefore predispose to CTS [20]. Third, sustained wrist flexion during infant feeding and care in the postpartum period increases carpal tunnel pressure and can provoke or worsen symptoms [3].

Diagnostics

Most classification systems for CTS integrate clinical symptoms, physical examination, and electrophysiological or ultrasonographic studies. In pregnant patients, diagnosis based on history and provocative tests is generally sufficient, since complete electrophysiological testing can be uncomfortable, and its routine use in this population is questionable. The omission of nerve conduction studies in pregnancy is often justified by the typically short, reversible nature of compression; however, electrodiagnostic testing may be warranted for atypical or persistent presentations. Ultrasonography offers a noninvasive alternative: Fowler et al.’s meta-analysis indicates ultrasound can serve as a feasible first confirmatory test, although electrodiagnostic studies remain the diagnostic gold standard [21].

Treatment

Given that PRCTS typically exhibits a milder clinical course than non-pregnancy CTS, characterized by mild to moderate symptoms of short duration with resolution shortly after delivery, conservative management is recommended [20]. Klein and Mondelli et al. reported that pregnant patients recover approximately three to four times faster than non-pregnant patients [22,23]. Because the pathogenic mechanisms underlying PRCTS are often reversible, surgical intervention is infrequently required and is reserved for cases with severe symptoms and functional impairment, failure of nonsurgical measures, and electrophysiological evidence of marked nerve compression [3,16].

**Figure 1:**
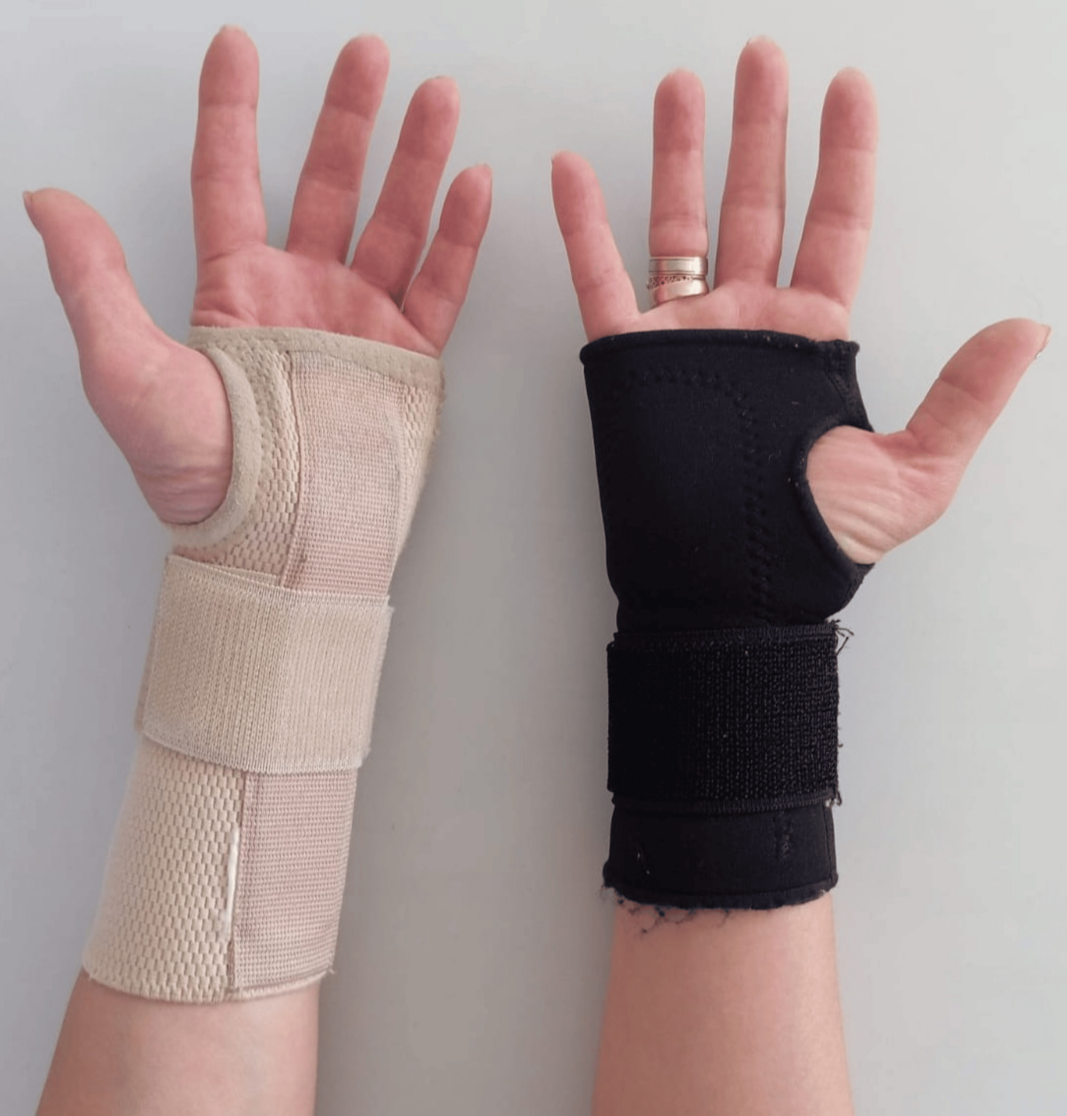
Volar-assisted and elastic wrist splints The attached figure is original and was prepared by the authors of the article. The elastic wrist splint is black; the volar-assisted orthosis is brown.

Conservative management of PRCTS focuses on reducing intracarpal pressure and includes wrist immobilization (education, orthoses, kinesiotaping), anti-edema strategies (e.g., corticosteroid injections, edema-reducing interventions), nerve-gliding exercises, and techniques to increase transverse carpal ligament (TCL) space (mobilization, myofascial release, deep-tissue massage) [3,15,16,20]. Patient education and activity modification emphasizing neutral wrist positioning, avoidance of repetitive stereotyped flexion, prolonged flexion, and forceful grips are integral components. Wrist orthoses represent a low-cost, well-tolerated intervention that typically does not require frequent clinical follow-up. Immobilization in a neutral to slight extension position (commonly 0-5° extension) reduces peak intracarpal pressure, improves local hemodynamics, diminishes swelling, and thereby alleviates ischemic symptoms [24-26]. Advantages include accessibility, simplicity, favorable tolerance, and minimal adverse effects. Orthoses are usually worn nocturnally for several weeks because the wrist tends to assume a flexed posture during sleep, increasing tunnel pressure; they may be used during daytime activities when symptoms are exacerbated [26]. Mete Cavus et al. reported clinical benefit with both volar-assisted and elastic wrist splints, with greater symptomatic improvement observed with elastic splints in PRCTS patients [20]. Abdelmenem et al. found that combining orthotic immobilization with nerve-gliding exercises significantly reduced symptom severity in pregnant women [27]. Figure 1 illustrates two orthosis types, volar-assisted and elastic wrist splints.

**Figure 2:**
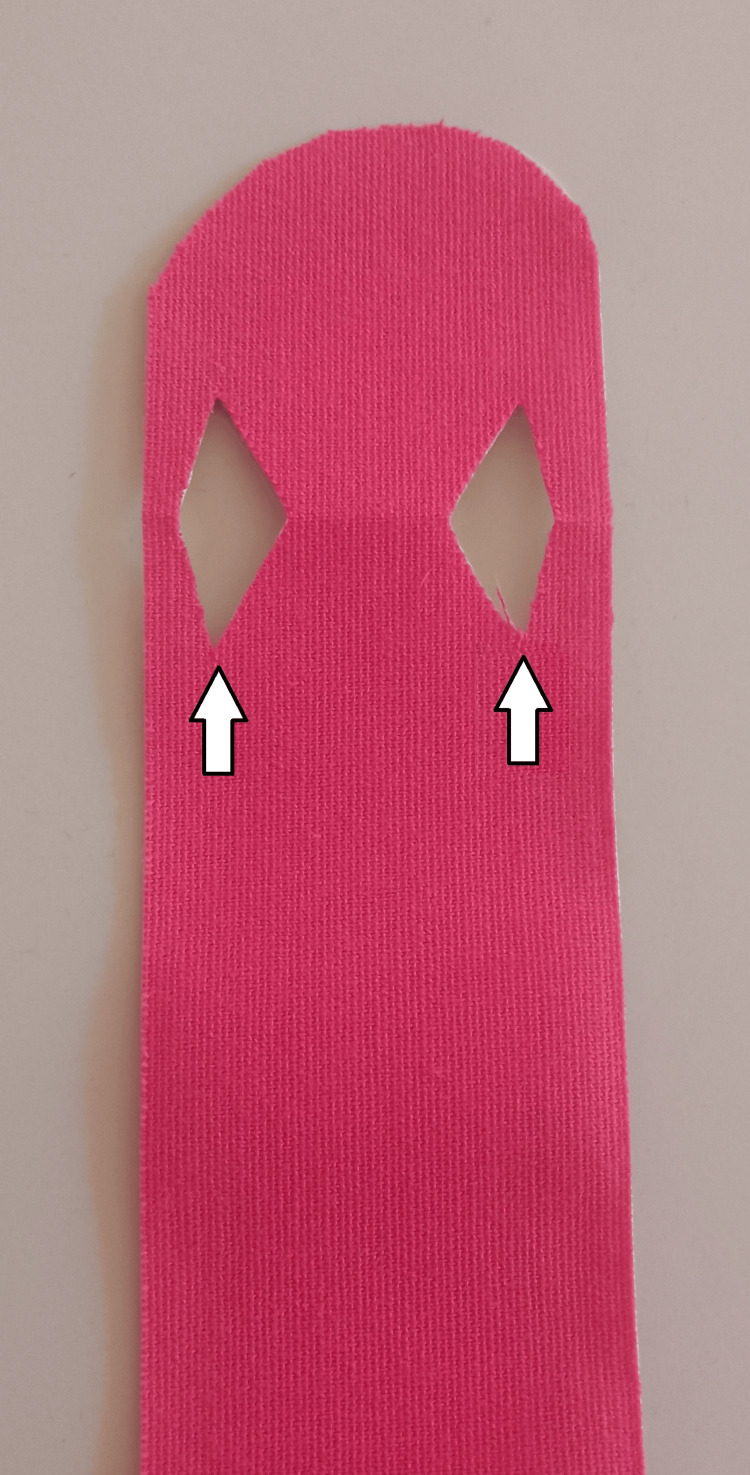
Preparing kinesiotape for application The attached figure is original and was prepared by the authors of the article. The arrows indicate longitudinal incisions that create separation for the third and fourth fingers.

**Figure 3:**
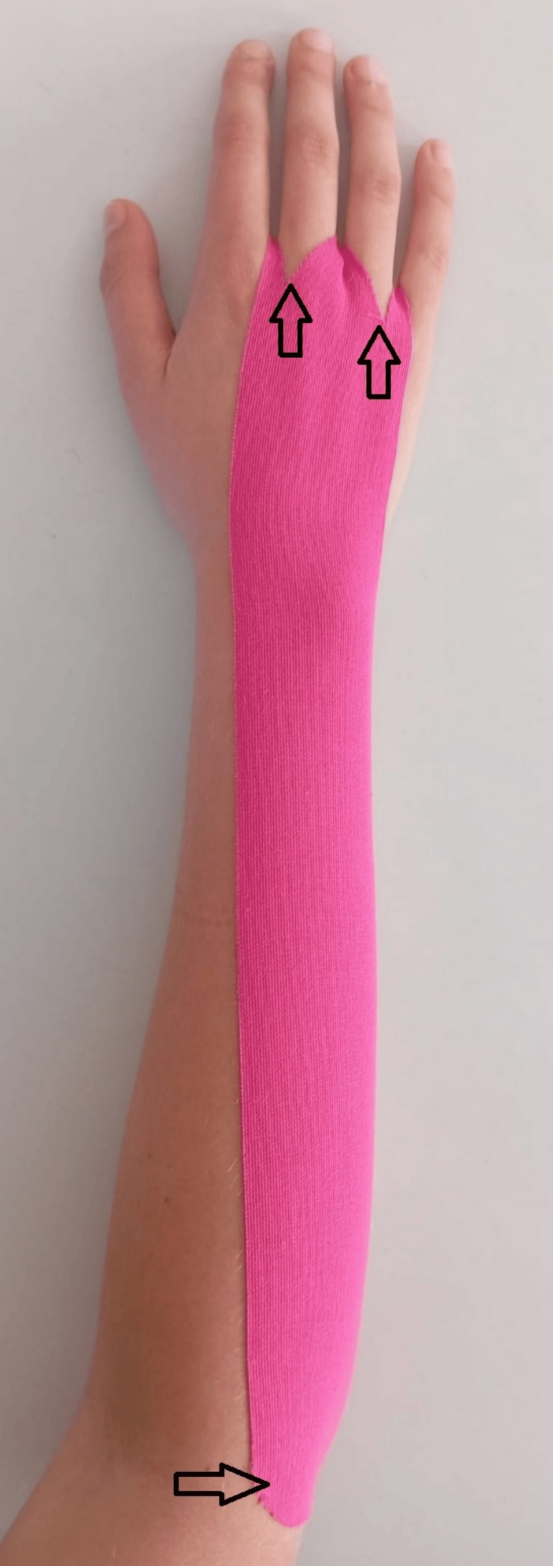
Kinesiotape application The attached figure is original and was prepared by the authors of the article. Tape is applied from the origin of the wrist extensors (arrow 1) to the dorsal aspects of the metacarpal bones. To improve adhesion and comfort, we recommend making longitudinal incisions that create separation for the third and fourth fingers (arrows 2 and 3).

An immobilizing effect may also be achieved with kinesiotaping, though its mechanism in CTS is not fully understood. In our practice, we use kinesiotape to approximate the effect of an orthosis: a tape is applied from the origin of the wrist extensors to the dorsal aspects of the metacarpal bones. To improve adhesion and comfort, we recommend making longitudinal incisions that create separation for the third and fourth fingers (Figures 2-3). A single, appropriately cut strip permits patients to self-apply the tape.

**Figure 4:**
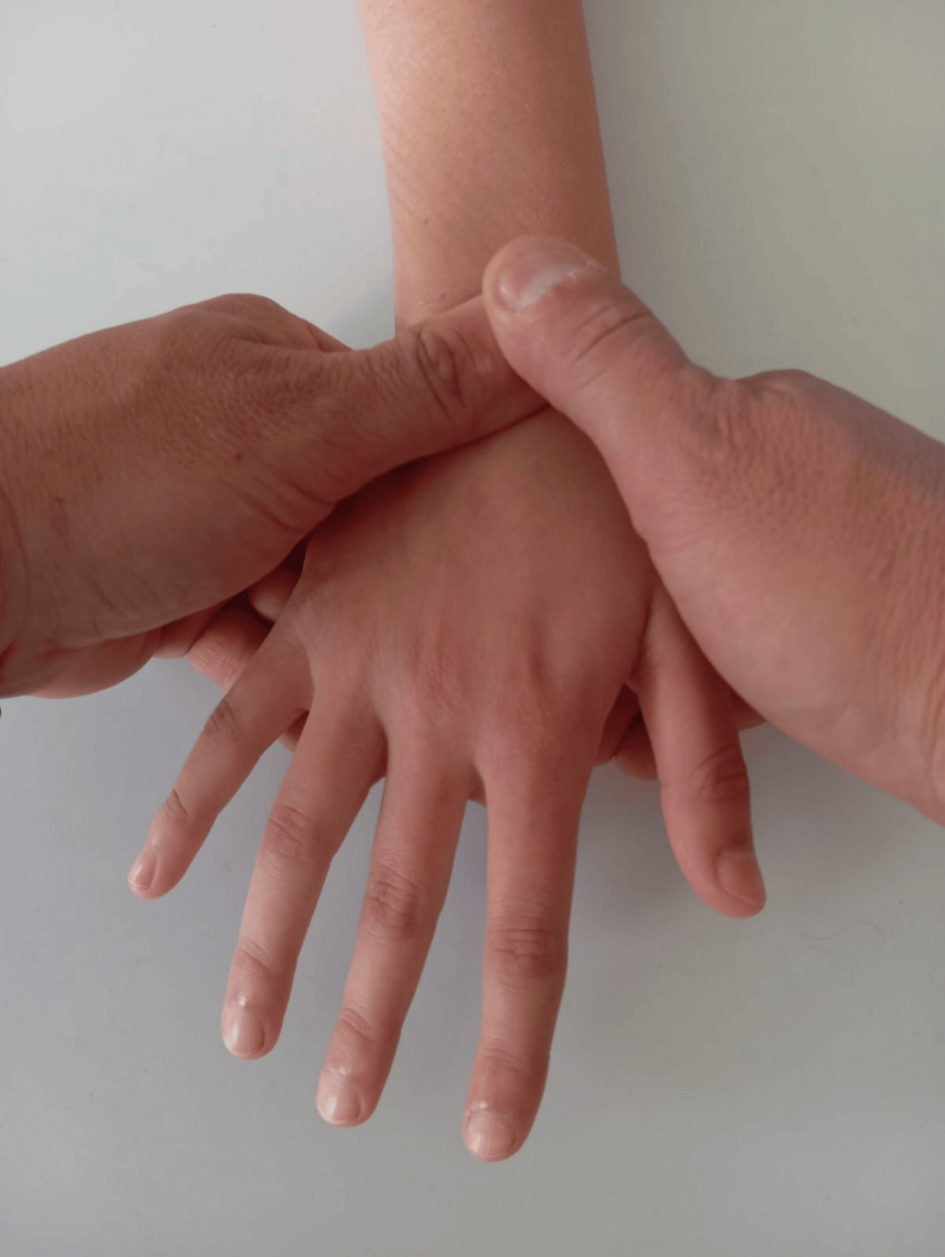
Mobilization of the transverse carpal ligament The attached figure is original and was prepared by the authors of the article. The therapist then applies simultaneous dorsal pressure with fingers II–III and palmar pressure on the lunate with the thumbs, aiming to “open” the carpal tunnel.

Corticosteroid injections are an effective option that reduces symptoms without adversely affecting the fetus or pregnancy when not contraindicated by maternal systemic disease [3,16]. In idiopathic CTS, the benefit is mainly anti-inflammatory; in pregnancy-related CTS, the predominant effect appears to be anti-edematous, lowering intracarpal pressure [24]. Morgan et al. also noted beneficial effects of steroids on surfactant production and lung maturation in preterm infants [28]. The common indication is acute symptom exacerbation in the third trimester, unresponsive to other measures. Dexamethasone acetate is often preferred in pregnancy for safety and reasonable symptom control [16]. Injections are usually given using anatomical landmarks to avoid injury to the median nerve and neurovascular structures; ultrasound guidance is increasingly used for real-time visualization to further reduce iatrogenic risk. Nerve and tendon-gliding exercises consist of sequential finger and upper-limb movements (including cervical mobilization) taught to the patient for home performance. When combined, these exercises help restore longitudinal nerve mobility, prevent adhesions between tendons and the median nerve, reduce swelling, improve venous return, and thus decrease intracarpal pressure. Their main advantage is safety and feasibility for independent home practice after instruction [27,29,30]. Stretching the transverse carpal ligament can be achieved with therapist-administered mobilization, self-mobilization, and deep-tissue massage. From about the eighteenth week of pregnancy, increased relaxin levels produce ligamentous laxity, which may facilitate TCL stretching and pressure reduction within the tunnel [16]. A described TCL mobilization technique places the therapist’s second and third fingers on the palmar surface near the distal half of the first metacarpal, the index and middle fingers of the other hand on the palmar surface near the pisiform and triquetrum, and both thumbs dorsally near the lunate. The therapist then applies simultaneous dorsal pressure with fingers II-III and palmar pressure on the lunate with the thumbs, aiming to “open” the carpal tunnel (Figure 4) [29].

**Figure 5:**
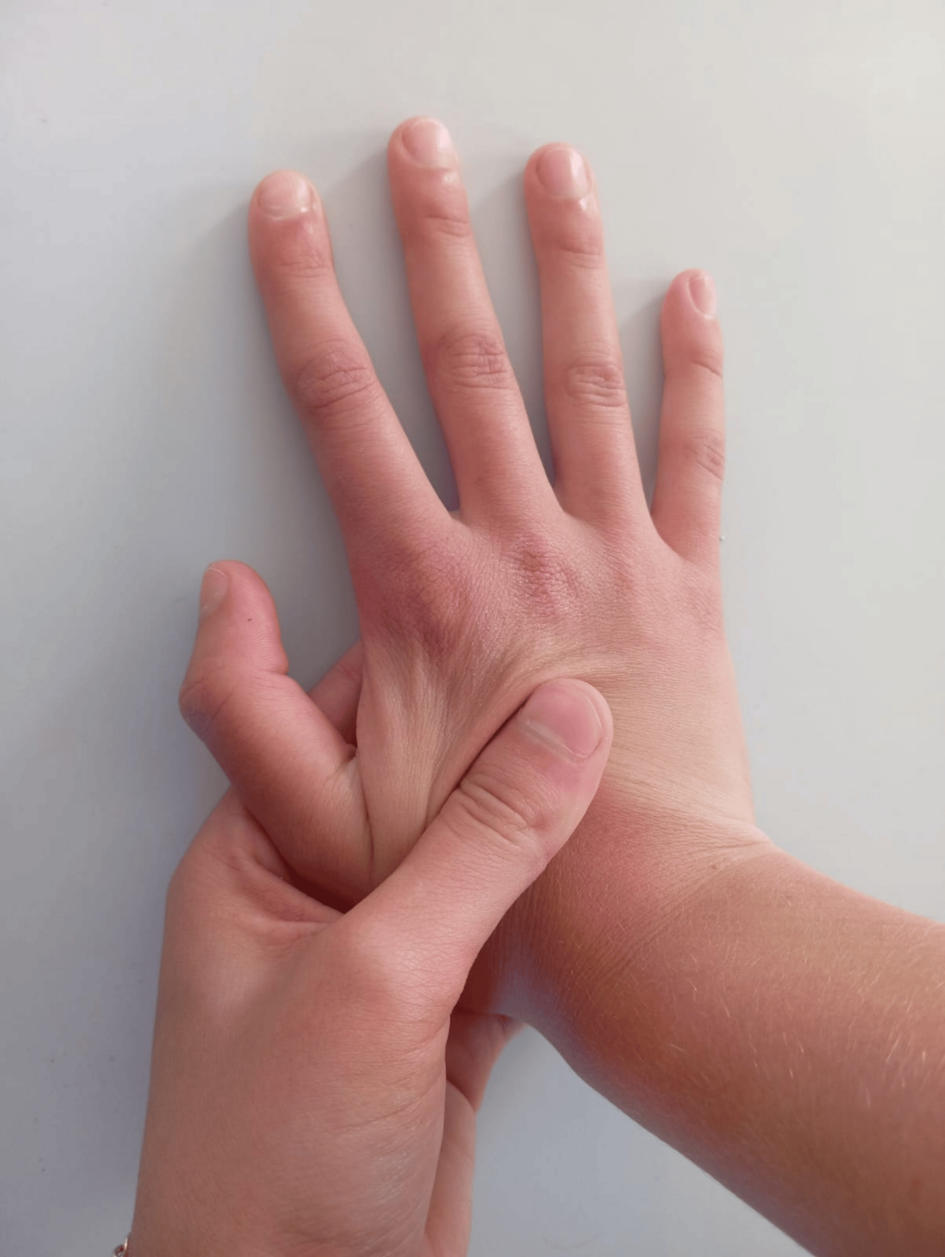
Automobilization of the transverse carpal ligament The attached figure is original and was prepared by the authors of the article. The patient places the wrist against a wall at approximately 90° and, with the contralateral hand, gently retracts the thenar eminence to stretch the transverse carpal ligament.

Passive stretching of the transverse carpal ligament can also be achieved by mechanical mobilization using devices such as the Dynasplint (Dynasplint Systems, Severna Park, Maryland, US) [31]. Shem et al. described a patient-assisted myofascial release technique: the patient places the wrist against a wall at approximately 90° and, with the contralateral hand, gently retracts the thenar eminence to stretch the transverse carpal ligament (Figure 5) [32].

Pharmacological treatment during pregnancy requires careful consideration because of potential maternal and fetal adverse effects. Drug therapy for PRCTS is reserved for symptom exacerbations or when conservative measures fail. In these situations, short-term use of non-steroidal anti-inflammatory drugs (NSAIDs) and analgesics may be permissible, but NSAIDs should be used cautiously, particularly after the thirtieth week of gestation. Acetaminophen (paracetamol) is the first-line analgesic for mild to moderate pain in pregnancy [14,16]. Physiotherapy options are similarly limited in pregnancy. Safer modalities that may be considered include low-level laser therapy, cryotherapy, and local thermotherapy. Modalities commonly used for CTS, such as high-intensity laser therapy, therapeutic ultrasound, phonophoresis, and extracorporeal shock wave therapy, are contraindicated or should be avoided during pregnancy, especially when their use carries the risk of affecting the patient's abdominal and pelvic areas.

Discussion

Because the symptoms of CTS often subside after childbirth, conservative management is preferred to avoid unnecessary interventions during pregnancy. Surgical intervention is infrequently required and is reserved for cases with severe symptoms and functional impairment, failure of nonsurgical measures, and electrophysiological evidence of marked nerve compression. Symptomatic patients can be treated using a wide range of physiotherapy techniques that are effective for the mother and safe for the fetus and are described in this review. The treatment methods often do not require specialized equipment. Some can be performed independently by the patient under periodic supervision or instruction from a physiotherapist (education and activity modification, orthoses, kinesiotaping, nerve-gliding exercises, and automobilization of the transverse carpal ligament).

Device manuals used in CTS therapy report that pregnancy is commonly listed as a contraindication. This applies to the following physiotherapy treatments: extracorporeal shockwave therapy, high-frequency laser therapy, ultrasound therapy, and phonophoresis. While reviewing the literature, we found articles and studies indicating that some of the procedures, which, in our opinion, should be contraindicated during pregnancy due to their unknown effects on the developing fetus, are being used. Due to the inconsistencies, we decided to review the literature. Laser therapy and extracorporeal shockwave therapy are widely regarded as contraindicated/avoided in pregnancy (or at least are listed as exclusion criteria in trials) [33,34]. Allameh et al. write that high-intensity laser therapy is considered an absolute contraindication in pregnant women and an exclusion criterion in some musculoskeletal studies. The North American Association for Laser Therapy conference has also recommended not using low-level laser directly over the developing fetus during pregnancy [33]. Cîmpeanu et al. consider the possibility of using high-frequency laser therapy, ultrasound therapy, iontophoresis, and phonophoresis [16]. Ashour et al. conducted a study using high-intensity laser therapy. Based on the research conducted, the authors state that high-intensity laser therapy, combined with a standard physical therapy program for CTS in pregnant women, is better than the physical therapy program alone to improve pain intensity and median nerve sensory distal latency [35]. Wilkerson et al. report that there are no randomized controlled trials in the literature on the safety of laser therapy during pregnancy. One review paper concluded that the available evidence, limited to low-evidence-level case reports and series, indicates cutaneous laser treatment during pregnancy is safe for both mother and fetus [36]. In the case of therapeutic ultrasound, guidance is mixed, especially when the procedure is administered away from the abdomen and pelvis. It seems reasonable to say that it is generally avoided or used only with caution/on a case-by-case basis, and away from the abdomen. Ghoraba et al. confirmed that phonophoresis is considered as the most efficient procedure that can be used to alleviate pain and reduce the inflammation and swelling associated with CTS during pregnancy. It is safe and has no side effects like medications [37].

In our opinion, using the above-mentioned forms of therapy for PRCTS is risky, despite their proven effectiveness in treating idiopathic CTS. Given that PRCTS symptoms are transient and resolve after delivery, it is probably not worth risking the health of the fetus when other highly effective conservative treatment methods are available, as described in this article. To further expand on the topic of conservative treatment for PRCTS, we conducted a literature review and presented the results in Table 2.

**Table 2:** Conservative treatment options

Authors	Treatment
Afshar, Tabrizi [3]	Activity modification, edema control, wrist splinting, and steroid injection
Ferraz et al. [14], Osterman [19]	Wrist splinting during sleeping and steroid injection
Ablove RH, Ablove TS [38]	Activity modification, wrist splinting, edema control, and steroid injections
Abdelmenem et al. [27]	Education, wrist splinting, hand and finger exercises, and heat treatment
Soad et al. [39]	Wrist splinting, especially at night, steroid injection, use of massage, and gentle exercise to provide muscle-pumping action
Hamoda et al. [13]	Neutral wrist splinting during sleeping; myofascial release technique: myofascial transverse carpal ligament release, interosseous membrane, and forearm muscles myofascial release
Mete Cavus et al. [20]	Wrist splinting, modification of activities of daily living; physical therapy: tendon-nerve shifting exercises, manipulation, acupuncture; pharmacological treatment: local steroid injection, nonsteroidal anti-inflammatory drugs, and vitamin B6
Cîmpeanu et al. [16]	Patient education, ergonomic modifications in the workplace, wrist splinting; physical therapy: stretching, pilates, occupational therapy, nerve and tendon gliding exercises, neuromuscular reeducation, kinesio taping, myofascial release therapy, mechanical traction; pharmacological treatment: non-steroidal anti-inflammatory drugs, analgesics, injectable corticosteroids, vitamin therapy; applying physiotherapy procedures: high-frequency laser therapy, ultrasound therapy, iontophoresis, phonophoresis, and hot-sand bath therapy

## Conclusions

In pregnancy-related carpal tunnel syndrome, the primary treatment goal is to reduce intracarpal pressure. First-line measures include wrist immobilization, pharmacologic and anti-edema therapies, and techniques to mobilize or stretch the transverse carpal ligament. Initiation of pharmacologic treatment during pregnancy should be carefully weighed because of potential maternal and fetal adverse effects. Commonly used physiotherapy procedures in the treatment of CTS, including high-intensity laser therapy and extracorporeal shock wave therapy, are contraindicated during pregnancy. Therapeutic ultrasound and phonophoresis are generally avoided or used only with caution. Surgical intervention is infrequently required and is reserved for cases with severe symptoms and functional impairment, failure of nonsurgical measures, and electrophysiological evidence of marked nerve compression. The conclusions presented in the article are not the result of a meta-analysis, and the data obtained were summarized descriptively.
